# Ox-LDL induced profound changes of small non-coding RNA in rat endothelial cells

**DOI:** 10.3389/fcvm.2023.1060719

**Published:** 2023-02-07

**Authors:** Yu Wang, Tianhua Liu, Wenying Xiao, Yanyan Bai, Dandan Yue, Liuliu Feng

**Affiliations:** Department of Cardiology, Shidong Hospital, Shidong Hospital Affiliated to University of Shanghai for Science and Technology, Shanghai, China

**Keywords:** endothelial cells, small non-coding RNA, piRNA, snoRNA, snRNA

## Abstract

**Introduction:**

Atherosclerosis (AS) is a common cardiovascular disease with a high incidence rate and mortality. Endothelial cell injury and dysfunction are early markers of AS. Oxidative low-density lipoprotein (Ox-LDL) is a key risk factor for the development of AS. Ox-LDL promotes endothelial cell apoptosis and induces inflammation and oxidative stress in endothelial cells. Small non-coding RNAs (sncRNAs) mainly include Piwi-interacting RNAs (piRNAs), small nucleolar RNAs (snoRNAs), small nuclear RNAs (snRNAs), microRNAs (miRNAs) and repeat-associated RNAs. Studies have shown that small non-coding RNAs play an increasingly important role in diseases.

**Methods:**

We used ox-LDL to treat rat endothelial cells to simulate endothelial cell injury. The expression changes of sncRNA were analyzed by small RNA high-throughput sequencing, and the expression changes of piRNA, snoRNA, snRNA, miRNA and repeat-associated RNA were verified by quantitative polymerase chain reaction (qPCR).

**Results:**

Small RNA sequencing showed that 42 piRNAs were upregulated and 38 piRNAs were downregulated in endothelial cells treated with ox-LDL. PiRNA DQ614630 promoted the apoptosis of endothelial cells. The snoRNA analysis results showed that 80 snoRNAs were upregulated and 68 snoRNAs were downregulated in endothelial cells with ox-LDL treatment, and snoRNA ENSRNOT00000079032.1 inhibited the apoptosis of endothelial cells. For snRNA, we found that 20 snRNAs were upregulated and 26 snRNAs were downregulated in endothelial cells with ox-LDL treatment, and snRNA ENSRNOT00000081005.1 increased the apoptosis of endothelial cells. Analysis of miRNAs indicated that 106 miRNAs were upregulated and 91 miRNAs were downregulated in endothelial cells with ox-LDL treatment, and miRNA rno-novel-136-mature promoted the apoptosis of endothelial cells. The repeat RNA analysis results showed that 4 repeat RNAs were upregulated and 6 repeat RNAs were downregulated in endothelial cells treated with ox-LDL.

**Discussion:**

This study first reported the expression changes of sncRNAs in endothelial cells with ox-LDL treatment, which provided new markers for the diagnosis and treatment of endothelial cell injury.

## Introduction

Atherosclerosis (AS) is a common cardiovascular disease with a high incidence rate and mortality (1, 2). It is the main cause of coronary heart disease, myocardial infarction and peripheral vascular disease. Endothelial cells play an important role in maintaining the dynamic balance of the vascular system (3). Their injury and dysfunction are early markers of AS. Stimulated by hypertension, hyperlipidemia and other factors, the adhesion molecules expressed by endothelial cells can promote the local accumulation of oxidized low-density lipoprotein (ox-LDL), induce a proinflammatory response, oxidative stress and endothelial cell apoptosis, and finally lead to the formation of plaques (4, 5). Elucidating the molecular mechanism of ox-LDL-mediated apoptosis of vascular endothelial cells (VECs) is of great significance to develop effective treatment methods for AS.

After LDL enters VECs, it combines with reactive oxygen species in cells to form ox-LDL (6). Ox-LDL causes damage to the structure and function of VECs, resulting in abnormal apoptosis of VECs (7). It can act as an apoptosis-inducing signal on the mitochondrial membrane, increase the permeability of the mitochondrial membrane, release the apoptosis promoter in mitochondria into the cytoplasm, improve the expression of the apoptosis-promoting gene Bax in cells, and activate the apoptosis initiating protease caspase-9, activating the downstream apoptotic effector protease caspase-3 through a cascade reaction, which acts on the cytoskeleton, resulting in apoptosis changes such as loss of DNA repair function and activation of endonuclease (8, 9). Ox-LDL passes through the gap between VECs to the subintima and combines with extracellular reactive oxygen species to form ox-LDL. At the same time, monocytes migrate through VECs to the subintima under the action of excessive inflammatory factors and are stimulated to turn into macrophages. The protective phagocytosis of macrophages makes the ox-LDL under the phagocytosis intima turn into foam cells, and the foam cells accumulate under the intima of the tube wall to form early lipid stripes of AS lesions (10–14). Therefore, elucidating the molecular mechanism of ox-LDL-mediated dysfunction of VECs is of great significance to develop effective treatment methods for AS.

Small non-coding RNAs (sncRNAs) mainly include Piwi-interacting RNAs (piRNAs), small nucleolar RNAs (snoRNAs), small nuclear RNAs (snRNAs), microRNAs (miRNAs) and repeat-associated RNAs (15). PiRNA is a type of small RNA with a length of approximately 30 nucleotides isolated from mammalian germ cells, and piRNA can play its regulatory role only when combined with members of the piwi protein family (16). The effect of piRNA on the growth and development of germ cells is regulated by gene silencing caused by the piwi piRNA complex (17). However, because research on piRNAs is still in the primary stage, some specific functions and biogenesis of piRNAs are still being studied. SnoRNA is a type of non-coding RNA with a length of 60-300 nucleotides that widely exists in eukaryotic cells (18). It is responsible for the post-transcriptional modification of other non-coding RNAs. SnoRNA can combine with specific proteins to form complexes to exist stably in cells. The main function of snoRNA is to guide the 2′-O-Ribose-Methylation and pseudouridylation modification of rRNA (19). SnRNA is a small molecule RNA containing approximately 50–200 nucleotides in the eukaryotic nucleus (20). The small ribonucleosome (snRNP) formed by its binding with related proteins mainly plays an important role in processing RNA precursors and removing excess fragments (such as introns). To date, miRNAs have been studied for more than 20 years, and their function involves the occurrence of a variety of diseases. Traditionally, miRNA plays a negative regulatory role in the cytoplasm by binding to the target gene 3′-UTR to inhibit the translation or degradation of mRNA. Studies have shown that sncRNA plays an increasingly important role in epigenetic regulation and is involved in the regulation of gene expression at many levels, such as gene transcription, post gene transcription and mRNA translation (21, 22). They are closely related to the diagnosis, treatment and prognosis of many human diseases (23–25). However, even though several reports have studied the role of miRNA in ox-LDL-induced endothelial injury (26, 27), until now, no investigation of piRNA, snoRNA, snRNA and repeat-associated RNA has been reported.

In this study, we used small RNA sequencing and quantitative polymerase chain reaction (qPCR) to analyze the effect of ox-LDL on the expression of sncRNAs in VECs and identified sncRNAs related to ox-LDL-induced VEC dysfunction, thus providing a new therapeutic target for the diagnosis and treatment of AS.

## Materials and methods

### Isolation and culture of rat aortic endothelial cells

The rats were killed after cervical dislocation, and the whole rats were soaked in 75% ethanol for disinfection. The chests were opened, and the aortas were dissected in sterile PBS. After excision of the fat and fibrous tissue of the vascular adventitia, the aortas were rinsed with PBS to remove the blood and coagulation in the vascular cavity. The aortas were then dissected into 1 mm^3^ arterial implants with a very sharp scalpel and planted in a culture dish. The intimal surfaces of the arterial grafts were contacted with the bottom of the culture dish, and the planting density was approximately 1 piece/cm^2^. The culture dish was placed in the incubator for 2 h. Culture medium containing 25% fetal bovine serum was added from the top of the tissue to slightly cover the intimal surface of arterial grafts. The plate was transferred into an incubator for 24 h, and after 24 h, the culture medium was changed. On the 3rd day, the endothelial cells were fully grown, and fibroblasts were just about to grow. Then, the arterial grafts were gently removed, and the culture medium was changed every 2 days.

### Ox-LDL treatment

The vascular endothelial cells were inoculated into the culture plate, 2 ml of medium containing 100 μg/ml ox-LDL was added to the culture plate for 12 h, and the cell samples were collected after 12 h of culture.

### RNA extraction

The lysed sample was placed at room temperature for 10 min to completely separate the nucleoprotein from the nucleic acid. Chloroform (0.2 ml) was added to the lysed sample, and the mixture was violently shaken for 15 s and placed at room temperature for 3 min. Then, the mixture was centrifuged at 12,000 rpm, 4°C, for 10 min. The upper aqueous phase was collected and mixed with an equal volume of isopropanol. The mixture was placed at room temperature for 20 min and then centrifuged at 12,000 rpm, 4°C, for 10 min. The supernatant was discarded, and 1 ml of 75% ethanol was added to wash the precipitate. RNA was purified after centrifugation at 12,000 rpm and 4°C for 3 min. Purified RNA was eluted in 30–50 μl of RNase-free ddH2O.

### Small RNA sequencing

A Qubit RNA detection kit was used to accurately quantify total RNA to determine the amount of total RNA added to the library construction. The 3′ end of RNA was connected to the connector with T4 RNA ligase 2. The 5′ end of RNA was connected to the connector with T4 RNA ligase 1. The connecting product was reverse-transcribed to obtain the cDNA strand of the connecting product. In this step, the small RNA library was reverse-transcribed into DNA. The reverse transcription product was amplified by PCR to obtain the final library product. The NextSeq 550 (Illumina) system was used for sequencing analysis. Raw reads were obtained after sequencing and then filtered, spliced and decontaminated to obtain clean reads, which were compared with the reference genome for subsequent information analysis. The data were removed with the original program. Clean data were obtained by removing low-quality sequences using the trimmatic program. The FastQC program was used to count the data volume of clean data. Fragments larger than 15 nucleotides were retained for subsequent analysis. The Bowtie program was used to compare clean data to the non-coding RNA database and genome. The edgeR program was used to analyze the differences in non-coding RNA expression. The databases used in this study were list as follow: PiRNABANK (piRNA), miRbase (miRNA) and Ensembl database (snoRNA and snRNA). As for repeat RNA were analyzed by RepeatMasker. The differentially expressed small RNAs are listed in Supplementary Table 1.

### qPCR

Total RNA was extracted from vascular endothelial cells by TRIzol. The EZ-press microRNA Reverse Transcription Kit was used for miRNA and piRNA reverse transcription. PrimeScript RT Master Mix was used for snoRNA and snRNA reverse transcription. SYBR Premix Ex Taq was used to detect the expression of sncRNA in VECs. U6 was used as the internal control. The relative expression of sncRNA was calculated by the 2^–ΔΔ^*^Ct^* method. The primers used are listed in Supplementary Table 2.

### SncRNA overexpression and knockdown

For piRNA and miRNA overexpression and knockdown, piRNA and miRNA mimics were used for overexpression, and piRNA and miRNA inhibitors were used for knockdown. The piRNA and miRNA mimics and inhibitors were designed and synthesized by General Biosystems (Anhui) Co., Ltd. A total of 50 μl of Opti MEM and 0.2 nmol of RNA were gently mixed to obtain RNA diluent. A total of 50 μl of Opti MEM and 2.0 μl of Lip2000 were gently mixed to obtain Lip2000 diluent, and the Lip2000 diluent was then incubated at room temperature for 5 min. RNA diluent and Lip2000 diluent were mixed and incubated for 20 min at room temperature to form the RNA-Lip2000 complex. RNA-Lip2000 complex was added to the incubated cells, and the culture plate was then gently shaken. Cells were incubated in a 37°C CO_2_ incubator for 4–6 h, and then the medium was changed and cultured for 18–48 h.

For snRNA and snoRNA overexpression, the target sequences of snRNA and snoRNA were cloned into the pLVX vector to construct lentivirus, which was used to transfect endothelial cells.

### Flow cytometry

Rat aortic endothelial cells were treated with ox-LDL for 12 h and then subjected to the indicated treatment. After that, cell apoptosis was measured using an Annexin V-FITC/propidium iodide kit (BD Biosciences) according to the manufacturer’s protocol. Apoptotic cells were observed using a flow cytometer. CellQuest software was used to analyze the apoptotic rate.

### Statistical analysis

All data are presented as the mean ± S.D. of three independent experiments. *T*-tests and ANOVA were used to compare two groups by Excel 2017; *p* < 0.05 was considered a significant difference, and *P* < 0.01 was considered highly statistically significant.

## Results

### Differential expression of piRNA in rat endothelial cells with ox-LDL treatment

**FIGURE 1:**
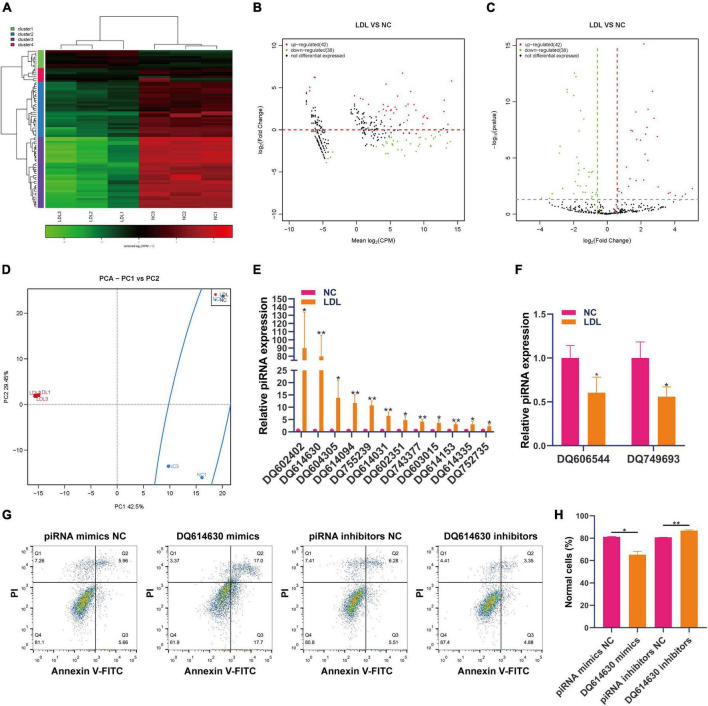
Differential expression of piRNAs in rat endothelial cells treated with ox-LDL. Heatmap **(A)**, MA map **(B)**, and volcano map **(C)** show the expression of piRNA in rat endothelial cells with ox-LDL treatment, *n* = 3. **(D)** PCA showed the expression of piRNA in rat endothelial cells with or without ox-LDL treatment, *n* = 3. **(E)** qPCR detected the expression of piRNA in rat endothelial cells with or without ox-LDL treatment. **(F)** qPCR detected the expression of piRNA in rat endothelial cells with or without ox-LDL treatment. **(G)** Flow cytometry analysis of cell apoptosis after treatment with piRNA DQ614630 mimics and piRNA DQ614630 inhibitors. **(H)** Quantification of normal cells according to flow cytometric analysis. For the statistical analysis, three independent experiments were conducted. *p < 0.05, **p < 0.01.

PiRNA is involved in transposon silencing, spermatogenesis, genome rearrangement, epigenetic regulation, protein regulation and reproductive stem cell maintenance (28). However, the regulatory effect of piRNAs on endothelial cell injury is unknown. Small RNA sequencing showed that 42 piRNAs were upregulated and 38 piRNAs were downregulated in ox-LDL-treated endothelial cells compared to NC cells (Figures 1A–C). We used PCA to show the relationship between piRNA expression and ox-LDL treatment. The obtained results showed that piRNA expression distinguished the ox-LDL group and NC group (Figure 1D). Next, we used qPCR to verify the small RNA sequencing results of piRNA. The qPCR results showed that compared with the NC group, the expression of 12 piRNAs in the ox-LDL group increased, and the expression of piRNA DQ602402 increased most significantly (Figure 1E). On the other hand, the expression of piRNA DQ606544 and DQ749693 was significantly decreased in the ox-LDL group compared to the NC group (Figure 1F). The expression of piRNA DQ614630 in endothelial cells was increased or knocked down by piRNA DQ614630 mimics or inhibitors (Supplementary Figure 1A). piRNA DQ614630 overexpression promoted the apoptosis of endothelial cells induced by ox-LDL, and piRNA DQ614630 knockdown inhibited the apoptosis of endothelial cells induced by ox-LDL (Figures 1G, H).

### Differential of snoRNA in rat endothelial cells with ox-LDL treatment

**FIGURE 2:**
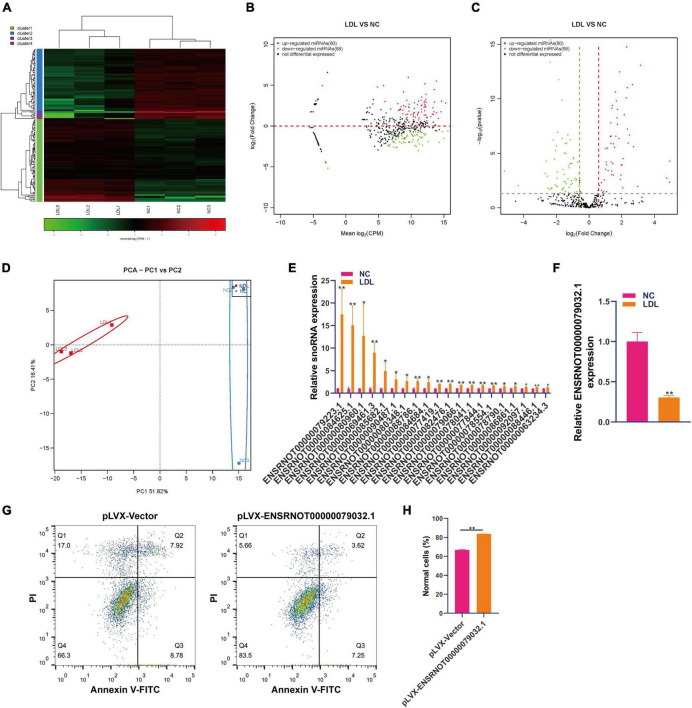
Differential expression of snoRNA in rat endothelial cells treated with ox-LDL. Heatmap **(A)**, MA map **(B)**, and volcano map **(C)** show the expression of snoRNA in rat endothelial cells with ox-LDL treatment, *n* = 3. **(D)** PCA showed the expression of snoRNA in rat endothelial cells with or without ox-LDL treatment, *n* = 3. **(E)** qPCR detected the expression of snoRNA in rat endothelial cells with or without ox-LDL treatment. **(F)** qPCR detected the expression of snoRNA in rat endothelial cells with or without ox-LDL treatment. **(G)** Flow cytometry assay of cell apoptosis after treatment with pLVX-ENSRNOT00000079032.1. **(H)** Quantification of normal cells according to flow cytometric analysis. For the statistical analysis, three independent experiments were conducted. *p < 0.05, **p < 0.01.

Research on snoRNA has developed rapidly, and recent studies have shown that snoRNA is also involved in genetic diseases, human variation, hematopoiesis, metabolism and cancer (19). Small RNA sequencing showed that 80 snoRNAs were upregulated and 68 snoRNAs were downregulated in ox-LDL-treated endothelial cells compared to NC cells (Figures 2A–C). The results of PCA indicated that snoRNA expression can distinguish the ox-LDL group and NC group (Figure 2D). qPCR analysis showed that compared with the NC group, the expression of 20 snoRNAs in the ox-LDL group increased, and the expression of snoRNA ENSRNOT00000079223.1 increased most significantly (Figure 2E). On the other hand, qPCR showed that the expression of ENSRNOT00000079032.1 was decreased in the ox-LDL group compared to the NC group (Figure 2F). The expression of snoRNA ENSRNOT00000079032.1 in endothelial cells was increased by transfection with lentivirus and detected by qPCR (Supplementary Figure 1B). snoRNA ENSRNOT00000079032.1 overexpression inhibited the apoptosis of endothelial cells induced by ox-LDL (Figures 2G, H).

### Differential expression of snRNA in rat endothelial cells with ox-LDL treatment

**FIGURE 3:**
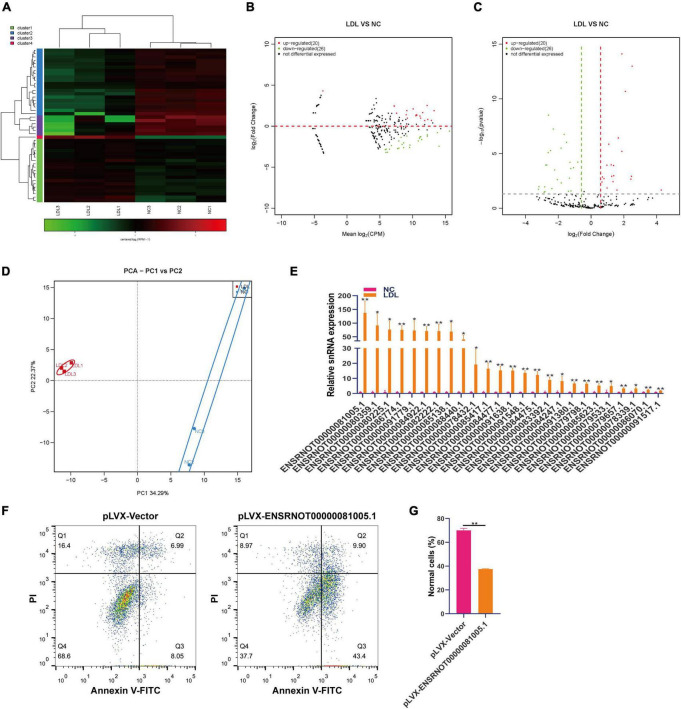
Differential expression of snRNA in rat endothelial cells treated with ox-LDL. Heatmap **(A)**, MA map **(B)**, and volcano map **(C)** show the expression of snRNA in rat endothelial cells with ox-LDL treatment, *n* = 3. **(D)** PCA showed the expression of snRNA in rat endothelial cells with or without ox-LDL treatment, *n* = 3. **(E)** qPCR detected the expression of snRNA in rat endothelial cells with or without ox-LDL treatment. **(F)** Flow cytometry assay of cell apoptosis after treatment with pLVX-ENSRNOT00000081005.1. **(G)** Quantification of normal cells according to flow cytometric analysis. For the statistical analysis, three independent experiments were conducted. *p < 0.05, **p < 0.01.

SnRNA includes a large group of non-coding RNAs with large differences, and these non-coding RNAs are mainly involved in the selective splicing of mRNA precursors and the processing of ribosomal RNA (29). We analyzed the expression changes of snoRNA in rat endothelial cells treated with ox-LDL. Small RNA sequencing showed that 20 snRNAs were upregulated and 26 snRNAs were downregulated in ox-LDL-treated endothelial cells (Figures 3A–C). PCA indicated that snRNA expression can distinguish the ox-LDL group and NC group (Figure 3D). We used qPCR to verify the small RNA sequencing results of snRNA. The qPCR results showed that compared with the NC group, the expression of 25 snRNAs in the ox-LDL group increased, and the expression of snRNA ENSRNOT00000081005.1 increased most significantly (Figure 3E). The expression of snRNA ENSRNOT00000081005.1 in endothelial cells was increased by transfection with lentivirus and detected by qPCR (Supplementary Figure 1C). snRNA ENSRNOT00000081005.1 overexpression inhibited the apoptosis of endothelial cells induced by ox-LDL (Figures 3F, G).

### Differential expression of miRNA in rat endothelial cells with ox-LDL treatment

**FIGURE 4:**
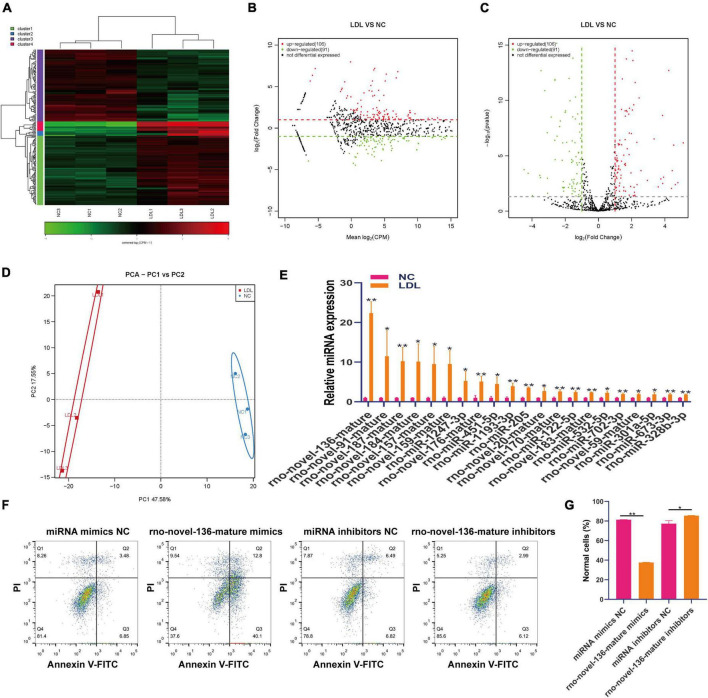
Differential expression of miRNA in rat endothelial cells treated with ox-LDL. Heatmap **(A)**, MA map **(B)**, and volcano map **(C)** show the expression of miRNA in rat endothelial cells with ox-LDL treatment, *n* = 3. **(D)** PCA showed the expression of miRNA in rat endothelial cells with or without ox-LDL treatment, *n* = 3. **(E)** qPCR detected the expression of miRNA in rat endothelial cells with or without ox-LDL treatment. **(F)** Flow cytometry assay of cell apoptosis after treatment with rno-novel-136-mature mimic and rno-novel-136-mature mimic inhibitors. **(G)** Quantification of normal cells according to flow cytometric analysis. For the statistical analysis, three independent experiments were conducted. *p < 0.05, **p < 0.01.

MiRNAs are involved in the maintenance of endothelial cell function (30, 31). Small RNA sequencing showed that 106 miRNAs were upregulated and 91 miRNAs were downregulated in ox-LDL-treated endothelial cells compared to NC cells (Figures 4A–C). We used PCA to show the relationship between miRNA expression and ox-LDL treatment. The obtained results showed that miRNA expression could distinguish the ox-LDL and NC groups (Figure 4D). qPCR analysis showed that compared with the NC group, the expression of 21 miRNAs in the ox-LDL group increased, and the expression of miRNA rno-novel-136-mature increased most significantly (Figure 4E). The expression of miRNA rno-novel-136-mature in endothelial cells was increased or knocked down by miRNA rno-novel-136-mature mimics or inhibitors (Supplementary Figure 1D). miRNA rno-novel-136-mature overexpression promoted the apoptosis of endothelial cells induced by ox-LDL, and miRNA rno-novel-136-mature knockdown inhibited the apoptosis of endothelial cells induced by ox-LDL (Figures 4F, G).

### Differential expression of repeat RNA in rat endothelial cells with ox-LDL treatment

**FIGURE 5:**
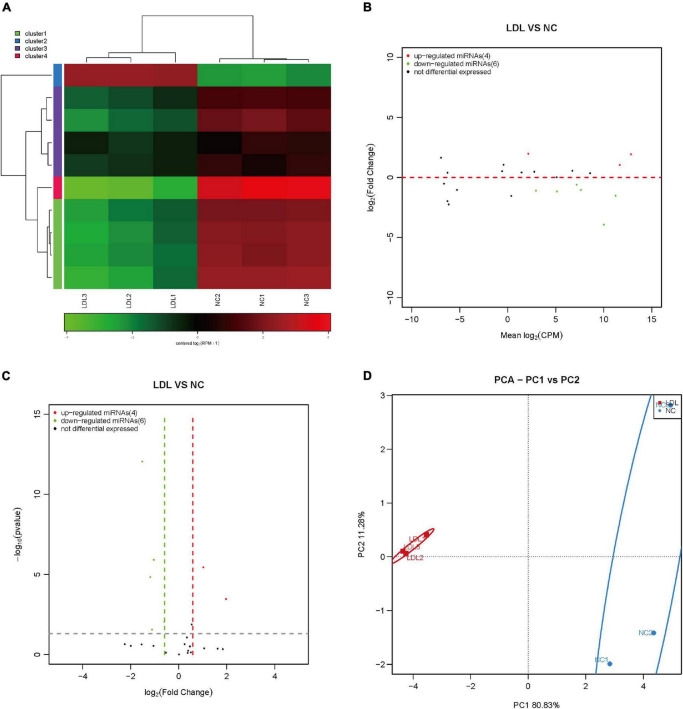
Differential expression of repeat RNA in rat endothelial cells treated with ox-LDL. Heatmap **(A)**, MA map **(B)**, and volcano map **(C)** show the expression of repeat RNA in rat endothelial cells with ox-LDL treatment, *n* = 3. **(D)** PCA showed the expression of repeat RNA in rat endothelial cells with or without ox-LDL treatment, *n* = 3.

All RNAs have a tendency to spontaneously fold into secondary or tertiary structures. Although RNA has only four types of nucleotides, they can form a variety of structures, thus affecting cell function. Aberrantly expanded, repeated RNA sequences can exhibit gain-of-function abnormalities and become pathogenic, giving rise to many diseases (32). The obtained results showed that 4 repeat RNAs were upregulated and 6 repeat RNAs were downregulated in ox-LDL-treated endothelial cells compared to NC cells (Figures 5A–C). PCA showed that repeat RNA expression could distinguish the ox-LDL group and NC group (Figure 5D).

## Discussion

The relationship between RNA structural and functional diversity in different organisms is the frontier of major basic research in life science. Especially with the proposal of genome and proteome projects, RNA omics research has become a new growth point of molecular biology research. In this study, we found the relationship between small RNAs and ox-LDL-induced ED by small RNA sequencing and qPCR. PiRNA is a type of small RNA isolated from mammalian germ cells, and piRNA can play its regulatory role only when combined with members of the piwi protein family (16). Currently, piRNAs have been detected in blood vessels and the heart, and previous studies have shown that piRNAs exert mechanistic regulatory potency during cardiac differentiation (33). PiRNA-30473 mediates the occurrence and prognosis of B lymphoma by regulating m6A methylation (34). PiRNA-DQ541777 mediates neuropathic pain through targeted regulation of Cdk5rap1 expression (35). PiRNA-63076 promotes the proliferation of pulmonary artery smooth muscle cells by regulating acyl-CoA dehydrogenase activity (36). However, no study has reported the regulatory effect of piRNAs on vascular endothelial cells. We first reported the effect of ox-LDL on the expression of piRNA in vascular endothelial cells, which may become a biomarker for the detection of vascular endothelial injury.

SnoRNA is a type of non-coding RNA with a length of 60–300 nucleotides that widely exists in eukaryotic cells (18). In vertebrates, the genes encoding snoRNAs mainly exist in the intron region of protein-coding genes or non-protein-coding genes and form mature snoRNAs after further posttranscriptional processing (18, 37). It can combine with specific proteins to form complexes to exist stably in cells. The main function of snoRNA is to guide the 2′-O-ribose-methylation and pseudouridylation modification of rRNA (19). Recent studies have shown that snoRNA is also involved in genetic diseases, human variation, hematopoiesis, metabolism and cancer (19). SNORD50A/B acts as a molecular switch to regulate the activity of the KRAS protein and then regulate the occurrence of tumors (38). Currently, no study has reported the expression changes of snoRNA in the process of endothelial cell injury caused by ox-LDL. We reported the expression changes of snoRNA in the process of endothelial cell injury caused by ox-LDL for the first time, which provides a new research and therapeutic target for the study of endothelial cell injury.

SnRNA is the main component of the RNA spliceosome in the posttranscriptional processing of eukaryotes (29). There are now five types of snRNAs with a length of approximately 100–215 nucleotides in mammals. snRNAs form RNA spliceosomes with approximately 40 nuclear proteins and play an important role in RNA posttranscriptional processing (29). The expression of snRNA in human, chimpanzee, rhesus monkey and mouse prefrontal cortices was systematically measured by high-throughput sequencing technology. Through comparative analysis, it was found that snRNA is very conserved at the gene family level, but the expression of U1 greatly changes in the human brain (39). We found that the expression of snRNA significantly changed during endothelial cell injury. However, the mechanism of snRNA needs to be further studied.

MiRNAs are the most studied sncRNAs, and their function is involved in almost all aspects of biological processes. An increasing number of studies have shown that miRNAs play an important regulatory role in cardiovascular diseases (40). Several reports have studied the role of miRNA in ox-LDL-induced endothelial injury. Mir-214-3p protects endothelial cells by targeting the expression of GPx4 (26). MiR-217 reduces ox-LDL-induced endothelial cell injury by inhibiting the expression of EGR1 (27). In this study, we found that some new miRNAs are related to endothelial cell damage caused by ox-LDL, and these new miRNAs need to be identified in sequence and function.

There are relatively few reports on the relationship between repeat-associated RNA and disease. Repeat-associated RNA was reported to be associated with myotonic dystrophy (DM), Fuch’s endothelial corneal dystrophy (FECD) and amyotrophic lateral sclerosis (ALS) (41). We first found expression changes in repeat-associated RNAs during ox-LDL-induced endothelial cell injury by small RNA sequencing.

Using small RNA high-throughput sequencing and qPCR detection, we found that the expression of piRNAs, snoRNAs, snRNAs, miRNAs and repeat-associated RNAs significantly changed during ox-LDL-induced endothelial cell injury. Our results preliminarily show that these sncRNAs are significantly upregulated or downregulated during ox-LDL treatment. PiRNA, snoRNA, snRNA, miRNA and repeat-associated RNA may be biomarkers of endothelial cell injury and could be useful in the future as therapeutic targets. Further research is still needed to reveal the cellular pathways regulated by sncRNAs that are involved in ox-LDL-induced endothelial cell injury.

## Data availability statement

The datasets presented in this study can be found in online repositories. The names of the repository/repositories and accession number(s) can be found in this article/Supplementary material.

## Ethics statement

This animal study was reviewed and approved by the Animal Ethics Committee of Shidong Hospital.

## Author contributions

YW wrote the manuscript, conducted the data analysis, and participated in the conception and conduction of the study. TL participated in the conception and conduction of the study and extensively engaged in the preparation and revision of the manuscript drafts. WX participated in the conception of the study, data analysis, and revision of the manuscript drafts. YB, DY, and LF participated in the conception of the study, supervised the conduction and data analysis, and extensively engaged in preparation and revision of the manuscript drafts. All authors contributed to the article and approved the submitted version.

## Supplementary material

Supplementary Figure 1 The overexpression and knockdown of ncRNA in rat endothelial cells. **(A)** The overexpression and knockdown of piRNA DQ614630 in rat endothelial cells. **(B)** The overexpression of ENSRNOT00000079032.1 in rat endothelial cells. **(C)** The overexpression of ENSRNOT00000081005.1 in rat endothelial cells. **(A,D)** The overexpression and knockdown of miRNA rno-novel-136-mature in rat endothelial cells. For the statistical analysis, three independent experiments were conducted. ^**^*p* < 0.01.
