# Supplementary figures and images for: Ox-LDL induced profound changes of small non-coding RNA in rat endothelial cells
Source: Front Cardiovasc Med. 2023 Feb 7;10:1060719. doi: 10.3389/fcvm.2023.1060719 (PMC9941181; doi:10.3389/fcvm.2023.1060719)

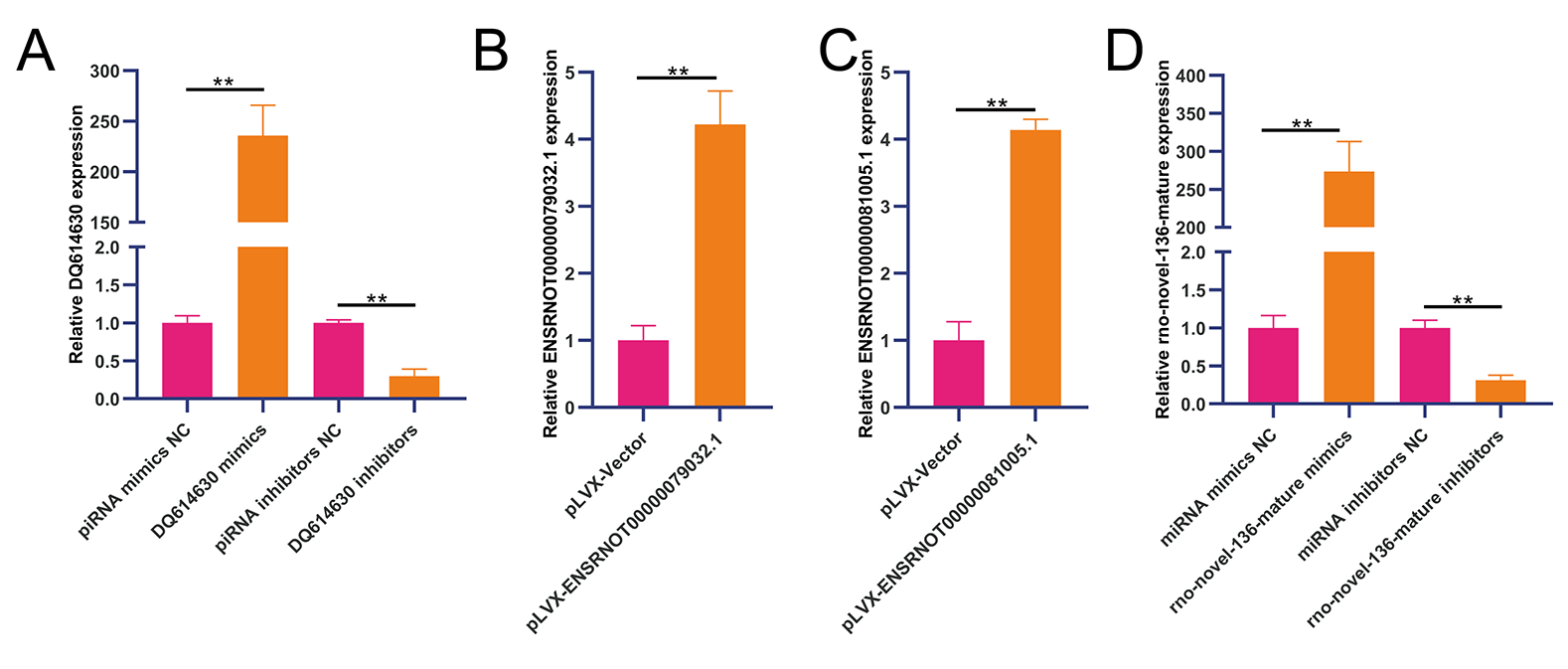

Supplement: Supplementary Figure 1 — The overexpression and knockdown of ncRNA in rat endothelial cells. (A) The overexpression and knockdown of piRNA DQ614630 in rat endothelial cells. (B) The overexpression of ENSRNOT00000079032.1 in rat endothelial cells. (C) The overexpression of ENSRNOT00000081005.1 in rat endothelial cells. (A,D) The overexpression and knockdown of miRNA rno-novel-136-mature in rat endothelial cells. For the statistical analysis, three independent experiments were conducted. **p < 0.01. [file Image_1.TIF]
